# Individual- and Herd-Level Milk ELISA Test Status and Incidence for Paratuberculosis in Hubei Province, China

**DOI:** 10.3390/vetsci11050202

**Published:** 2024-05-07

**Authors:** Yingyu Chen, Liyue Hou, Abdul Karim Khalid, Ian Duncan Robertson, Yuhao Zhao, Xi Chen, Aizhen Guo

**Affiliations:** 1 National Key Laboratory of Agricultural Microbiology, College of Veterinary Medicine, Huazhong Agricultural University, Wuhan 430070, China; chenyingyu@mail.hzau.edu.cn (Y.C.); hly0825@foxmail.com (L.H.); drabdulkarim43@webmail.hzau.edu.cn (A.K.K.); zhaoyuhao@webmail.hzau.edu.cn (Y.Z.); chenxi@mail.hzau.edu.cn (X.C.); 2 National Animal Tuberculosis Para-Reference Laboratory (Wuhan), Hubei International Scientific and Technological Cooperation Base of Veterinary Epidemiology, Huazhong Agricultural University, Wuhan 430070, China; i.robertson@murdoch.edu.au; 3 School of Veterinary Medicine, Murdoch University, Murdoch, WA 6150, Australia

**Keywords:** bovine paratuberculosis, milk antibody ELISA, prevalence, incidence, milk quality

## Abstract

**Simple Summary:**

Bovine paratuberculosis, a chronic infectious disease, is a significant concern in ruminants and wild animals, often leading to severe health issues and economic losses. In China, it is a prevalent yet underreported bovine disease. Our study aimed to fill this gap by investigating the prevalence of bovine paratuberculosis in Hubei Province. We also evaluated milk and blood antibody tests for paratuberculosis, which showed a high overall agreement of 92.0%. Using the milk test, we found that the highest lacto-prevalence at the individual level reached 22.9%, with a farm-level prevalence of 92.3% in January and 84.6% in April 2018. The total incidence risk of all farms was 6% per 3 months. Notably, large-scale farms had a significantly lower prevalence and incidence than small-scale farms. These findings underscore the need for robust prevention and control measures. Furthermore, our study underscores the significant impact of bovine paratuberculosis on milk quality, with a notable increase in somatic cell counts. These findings provide crucial insights into the prevalence and incidence risk of paratuberculosis in China, serving as a vital foundation for advocating for its prevention and control.

**Abstract:**

*Mycobacterium avium* subsp. *paratuberculosis* (MAP) is responsible for the persistent infectious illness known as bovine paratuberculosis, which is one of the most easily overlooked diseases in China amid a lack of epidemiological data. In this study, we evaluated the agreement of milk and blood antibody tests for paratuberculosis and showed an overall agreement of 92.0%, with a 95.0% negative coincidence rate and a 78.6% positive coincidence rate. The milk test was then used to examine the prevalence and incidence of dairy cows in Hubei Province, China. We found that, at the individual level, the highest lacto-prevalence reached up to 22.9%; the farm-level prevalence was as high as 92.3% (12/13) and 84.6% (11/13) in January and April 2018, respectively. The total incidence risk of all farms was 6% per three months. We also found that large-scale farms had a significantly lower prevalence and incidence than small-scale farms. Finally, the correlation between paratuberculosis and milk quality was evaluated, and we confirmed that MAP can significantly alter milk quality and raise somatic cell counts in the milk. This study provides valuable information for assessing the prevalence and incidence risk of paratuberculosis in China. It further provides an essential basis for calling for the prevention and control of paratuberculosis in China.

## 1. Introduction

Bovine paratuberculosis, often known as Johne’s disease, is a chronic infectious disease found in both wild animals and ruminants such as cattle, sheep, goats, deer, camelids, and primates [1] and caused by *Mycobacterium avium* subsp. *paratuberculosis* (MAP) [2], a Gram-positive and also acid-fast bacterium that belongs to the *Mycobacterium avium complex,* which usually results in intestinal infection [3]. It can be transmitted both horizontally and vertically with a long incubation time. Paratuberculosis-infected animals usually have slight clinical symptoms, which very easily lead to immunosuppression and a higher risk of susceptibility to infection with other diseases [4]. As a long-lasting disease, MAP-infected animals can often be affected by granulomatous enteritis, diarrhea, weight loss, hypoproteinemia, emaciation, a decline in milk supply, a decrease in reproductive ability, and ultimately mortality [5,6]. Globally, MAP leads to losses of more than USD 1.5 billion each year and annual economic losses of USD 21–79 per cow [6,7]. In the US, the economic losses are about USD 198.42 million; in Europe, the losses are around USD 364.31 million [6]. As most MAP-infected cows do not exhibit clinical signs at the early stage [8], it is one of China’s most easily overlooked bovine diseases.

Paratuberculosis is endemic in many parts of the world [9]. Previous studies reported that in most countries with substantial dairy businesses, the prevalence of MAP infection at the herd level is expected to reach 50% [10]. In Western Uganda, the real animal-level prevalence was more than 3%, with a herd-level prevalence of more than 40% [11]. In Ethiopia, on the basis of the prevalence of gross lesions, the prevalence even reached 11.25% [12]. In Brazil, in the high-producing dairy herds, using qPCR to test the fecal samples, the animal prevalence was 9.8% and the herd-level prevalence was 61.1% [13]. Sporadic reports showed that the current situation of paratuberculosis in China is also severe in some areas. A meta-analysis based on five databases showed that the average animal prevalence of paratuberculosis was 6.95% in China [14]. However, different regions had a different prevalence. In northern and northeastern China, the seroprevalence of MAP was 11.79%, with a 20.35% herd-level seroprevalence; in Shandong Province, the herd prevalence was about 60%, and the animal level was around 10% [15,16]; and in Inner Mongolia, the prevalence was 10.79%, based on serum tests [17]. This highlights the widespread nature of the disease within dairy cattle populations and underscores the urgent need for effective control and management strategies in China.

Prompt and reliable diagnosis is essential for preventing the spread of the disease, implementing herd management strategies, and minimizing financial losses [18]. Traditional fecal culture is the gold standard for diagnosing paratuberculosis, but it is labor-intensive, time-consuming, and less sensitive, particularly in the early stages of infection [19]. In this context, serological tests evaluating the humoral response are often used to detect MAP-specific antibodies [20]. They have a low cost, high specificity and sensitivity, ease of implementation, and speed. Quantitative research reported that milk samples could replace serum samples in identifying infected herds by an antibody ELISA [21,22]. Milk-based ELISA testing has various benefits, including ease of use, a reduced impact on animal welfare, and accessibility due to frequent milk collection [23], and it ensures the animals’ safety and minimizes occupational hazards for farm workers. It was reported that, globally, 1–3% of milk tested positive in cows either clinically or subclinically infected with MAP [2]. As a result, incorporating ELISA testing into milk samples seamlessly adds to herd health surveillance and monitoring. This integration allows for more efficient and frequent testing, facilitating the early detection and prompt management of MAP infections [24]. 

As experienced experts have said, understanding the relationship between paratuberculosis and milk quality is strongly related to the farmers’ attitude toward paratuberculosis prevention and control. Although the economic impact of paratuberculosis in dairy cattle has been the focus of many studies in different counties [8], to our knowledge, the evaluation has yet to be done in China. 

In this current study, we analyzed the agreement of serum- and milk-based ELISA tests for paratuberculosis, evaluated the prevalence and incidence of paratuberculosis, and finally evaluated the association between lacto-prevalence and milk quality in 15 commercial dairy farms in Hubei Province, China. By introducing this approach, we seek to enhance the accuracy and efficiency of the diagnosis of MAP, ultimately improving the control and management of this infectious disease.

## 2. Materials and Methods

### 2.1. Study Area and Samples

The blood and milk samples were taken from 149 randomly selected dairy cows from three dairy farms (32 and 83 from two small-scale farms and 34 from a large-scale farm) in Hubei Province to evaluate the agreement of the milk and serum antibody ELISA tests. 

For the lacto-prevalence detection, we randomly selected 1596 milk samples in total from 12 dairy cattle farms in January 2018, and 1256 milk samples were collected from 13 dairy farms in April 2018. Among these samples, 869 cows were tested twice. There were 69 cows that were lacto-prevalence positive at the beginning. Those dairy cows were removed from the subgroup, and the remaining 800 cows were used to calculate the incidence risk.

The farms were categorized into large farms (where the number of lactating cows ≥ 1000) and small farms (where the number of lactating cows < 1000) in three cities of Hubei Province. The location, scale, and sampling information from the different farms where the samples were collected are presented in Table 1. No prior information was available on the prevalence of paratuberculosis on the sampled farms.

### 2.2. Milk and Serum Antibody Tests for Paratuberculosis

Both the milk and blood samples were treated as previously reported [25]. Briefly, for the milk samples, the whole-fat milk samples were stored at 4 °C overnight, the supernatant was discarded, and the (whey) residue was kept for further use at −20 °C. All blood samples were stored at room temperature for 1–2 h and centrifuged at 4000 rpm for 10 min, and the supernatant was collected and stored at −20 °C for further use. The antibody test kit (*Mycobacterium paratuberculosis* Antibody Test Kit), which can be used for both milk and serum antibody tests, was purchased from IDEXX Laboratories, Inc. (Westbrook, ME, USA) Both tests were conducted and interpreted by the manufacturer’s guidelines.

### 2.3. Milk Quality and Paratuberculosis Antibody Correlation

All data and information concerning the milk yield and quality, including the percentages of fat, protein, lactose, total solids, and urea nitrogen in the milk and the somatic cell counts (SCCs, ×1000 cell/mL), were provided by DHI by using the CombiFoss FT + milk composition and somatic cell analyzer, as previously described [23]. The complete information on both the milk quality and the milk antibody tests were available for 1596 and 1256 individual animals from 12 and 13 cow farms in January and April 2018, respectively. Among those cows, 1061 had been evaluated just once and 895 twice (a total of 1956 cows and 2852 milk samples). 

### 2.4. Statistical Analysis

Statistical analysis with Cohen’s kappa was used to check the coincidence of the serum and milk antibody tests using the online Epitools (https://epitools.ausvet.com.au/comparetwotests) software (accessed on 1 May 2018). Where kappa = 1, perfect agreement was indicated, and, where kappa = 0, the agreement was the same as would be expected by chance. Where kappa < 0, there was a weaker agreement than expected by chance. The higher the kappa, the stronger the agreement and the more dependable it was. The relative risk was calculated according to [26]. The risk, which uses “new cases divided by the undiseased animal at the very beginning”, was calculated using the method of [27]. The Kolmogorov–Smirnov test was used to confirm whether the data were normally distributed or not. A *t*-test was used to evaluate the significance of lacto-prevalence on the milk quality (mean ± SD).

### 2.5. Ethics Approval

The Huazhong Agricultural University Animal Experimental Ethics Committee approved the animal experiment plan under protocol number HZAUCA-2019-006.

## 3. Results

### 3.1. Coincidence Rate between Milk and Serum Antibody ELISA Tests

A total of 149 paired milk and blood samples were collected and tested with the ELISAs. Overall, 30 out of 149 cows tested positive using the serum antibody test (the reference test), among which 22 (73.3%, 95% CI: 54.1, 87.7) also tested positive using the milk antibody ELISA; of the remaining 119 serum antibody test negative cows, 115 (96.6%, 95% CI: 91.6, 99.1) also tested negative with the milk antibody ELISA (Table 2). 

The overall agreement between the milk and serum ELISAs was 92.0% (95% CI: 86.4, 95.8), with a higher negative coincidence rate (95.0%, 95% CI: 91.5, 97.4) than a positive coincidence rate (78.6%, 95% CI: 65.6, 88.4). The kappa value was 0.736 (*p* < 0.001), which showed that the milk antibody test had a good agreement with the serum antibody test.

### 3.2. Lacto-Prevalence of MAP at Different Farms

In January 2018, 1596 milk samples were randomly collected from 12 dairy farms, and 1256 milk samples were collected from 13 dairy farms in April 2018.

Overall, 5 out of 13 (38.4%) farms had a higher than 10% prevalence in both sampling periods, and 2 out of 13 (15.4%) farms had a higher than 10% prevalence once. In total, 3 out of 13 (23.1%) farms had a less than 3% prevalence in both tests, and only 2 out of 13 (15.4%) farms presented a 0% prevalence once.

With respect to the different time periods, in January 2018, 132 out of 1596 samples tested positive, with an overall lacto-prevalence of 8.3% (95% CI: 7.0, 9.7). The highest test prevalence was 22.9% (95% CI: 15.0, 32.6) in Farm L, followed by 18.8% (95% CI: 10.9, 29.0) in Farm D, 18.2% (95% CI: 10.3, 28.6) in Farm B, and 17.1% (95% CI: 11.3, 24.4) in Farm J. 

The overall lacto-prevalence in April 2018 was 9.8% (95% CI: 8.2, 11.6), with the highest test prevalence of 19.9% (95% CI: 13.7, 27.3) in Farm C (which was not tested in January 2018), followed by 18.7% (95% CI: 10.6, 29.3) in Farm L, which had the highest test prevalence in January, then 17.6% (95% CI: 8.4, 30.9) in Farm K. Farm G had the lowest lactoprevalence in both tests: 1.3% (95% CI: 0.2, 4.6) in the first test and 0 (95% CI: 0.0, 3.9) in the second test (Table 3). 

At the farm level, in January, all 12 farms tested positive with a 100% (12/12) (95% CI: 73.5, 100) lacto-prevalence; in April 2018, 84.6% (11/13) (95% CI: 54.6, 98.1) of the herd tested positive. This indicated that paratuberculosis was presenting a very serious epidemic status in Hubei province.

The farms were also categorized into large and small farms in three cities (WH, HG, and YC) of Hubei Province. The test prevalence for the large farms was 4.1% (95% CI: 2.8, 5.8) and 4.4% (95% CI: 2.7, 6.6) in January and April, respectively, which was significantly lower than the small farms where the test prevalence was 12.2% (95% CI: 10.0, 14.6) and 12.9% (95% CI: 10.7, 15.5) (Table 3). This indicated that the small-scale farms had a relatively higher risk than that in the large-scale farms (RR = 2.97, 95% CI: 2.21, 3.99) on average. 

### 3.3. Incidence Risk of MAP at Different Farms

The incidence risk of paratuberculosis was also calculated for the sampled animals in Hubei Province. The total incidence risk of all farms was 6% per 3 months (95% CI: 4.5, 7.9). Farms I and D had the highest incidence risk of 15.2% per 3 months (95% CI: 6.3, 28.9 and 5.1, 31.9), followed by Farm J with 14.9% (95% CI: 7.7, 25.0) and Farm M with 10.3% (95% CI: 4.5, 19.2). Farms A and G had the lowest incidence risks of 0.0% (95% CI: 0.0, 3.7, and 0.0, 4.1, respectively) per 3 months. 

Furthermore, we calculated the incidence risk of both the large and small farms, and we found that the total incidence risk for lactating cows in the large-scale dairy farms in Hubei Province was 2.8% (95% CI: 1.3, 5.2) per 3 months, significantly lower than the small-scale farms where it was 8.2% (95% CI: 5.9, 11.1) (*p* < 0.05) (Table 4). This indicated that the dairy cattle in small-scale farms were more likely to be infected with MAP compared with in large-scale farms.

### 3.4. Relationship between Bovine Paratuberculosis and Milk Quality

In order to detect the impact of paratuberculosis on milk production and quality, we compared the milk yield, fat content percentage, milk protein percentage, lactose content, total solids percentage, SCCs, and urea nitrogen percentage in the positive lacto tests and negative milk samples. The results showed that the animals that were positive on the milk test for paratuberculosis had a 14.2% reduction in daily milk production, a 4% loss in lactose content, a 5.5% increase in milk protein content, and as high as a 53.9% increase in the somatic cell count (SCC). There was no significant difference between the negative and positive cows for the milk fat percentage, total solids, and urea nitrogen (Table 5).

## 4. Discussion

Few reports exist on the epidemiology of paratuberculosis in dairy cows in China and the evaluation of paratuberculosis and milk quality. In this study, we evaluated the serum- and milk-based ELISA tests for detecting MAP and tested the prevalence and incidence risk in dairy cows in Hubei Province, China. We further assessed the correlation between the paratuberculosis antibody levels and milk quality.

The accurate and timely diagnosis of paratuberculosis is crucial for the effective management and control of the disease. MAP is a bacterium that grows slowly, with a generation time of more than 24 h, with 7–16 weeks usually being needed for the isolation, due to a low sensitivity. Therefore, culture and isolation is not suitable for the detection of MAP in the field [28]. The typical test for paratuberculosis is a blood-based ELISA, usually a serum antibody test. Compared with isolation of the bacteria, an ELISA needs less time and is much more easy to conduct, with a higher sensitivity (reported as 84.3%) and specificity (96.6%) [29]. A critical distinction to be considered is that the procurement of blood samples necessitates the employment of invasive methodologies, including venipuncture. Such procedures have been documented to elicit stress responses and inflict discomfort on the animals [23]. Conversely, the acquisition of milk samples is an integral and customary practice in dairy farming. It incorporates non-invasive methodologies that impose minimal stress on animals, thus providing a more ethical alternative. Milk antibody tests are widely used in the detection of many diseases, such as bovine tuberculosis [23], Q fever [30], *Mycoplasmopsis* *bovis* [31], and the bovine gammaherpesvirus 4 [32]. These findings coalesce to underscore the utility of a milk-based ELISA as a productive and viable alternative to serum-based assays for detecting MAP and bTB in dairy cattle. This study advocates for the judicious integration of milk ELISAs within the surveillance and diagnostic frameworks that are used to manage and contain these infections.

This current study found that serum- and milk-based ELISAs for paratuberculosis had a coincidence rate of 92.0% (95% CI: 86.4%, 95.8%). This aligns with previous studies [33], which indicated that a milk antibody ELISA for paratuberculosis is highly recommended as the complement method in paratuberculosis surveillance. Not only with a high agreement with the serum ELISA, the milk ELISA has also been proven to have a good consistency with the MAP culture [34]. It is not only individual tests but also bulk tank milk that is widely used in cattle disease testing or even surveillance [30,31]. Although we did not evaluate the ability of bulk tank milk testing in this study, based on previous studies, it should have good application prospects in the detection of paratuberculosis.

Furthermore, this present investigation utilized milk antibody assays to ascertain the prevalence of MAP at both the individual animal and herd strata within the geographic confines of Hubei Province. The results revealed an alarming endemic of bovine paratuberculosis in Hubei Province (Table 3), with a high prevalence of 8.3% (95% CI: 7.0, 9.7%) to 9.8% (95% CI: 8.2, 11.6). At the herd level, the prevalence of MAP was even more concerning, reaching 92.3% (95% CI: 64, 99.8) during the first sampling and 84.6% (95% CI: 54.6, 98.1) during the second sampling (Table 3). These findings are partly in concordance with other studies. In Shandong Province, the herd prevalence was 57.9%, and 11.7% of animals tested seropositive for MAP [16]. In Xinjiang, the overall apparent prevalence of MAP was 4.8% at the individual level and 50.0% at the herd level [35]; in intensive farming herds, the herd prevalence reached 88.9% [35]. 

The incidence risk of paratuberculosis was also calculated in our study (Table 4). To our knowledge, this is the first study investigating the paratuberculosis incidence risk in China. Our data showed a 6% incidence risk per 3 months (95% CI: 4.5, 7.9). But some farms had a risk as high as more than 10% per 3 months, which means that, if no control measures were taken, 6–10% animals in a dairy herd could be infected with MAP in 3 months. Hubei is located in the middle of China, which is an important geographical location, and both the prevalence and incidence risk raise concerns that, without intervention, MAP will continue to spread from Hubei to other parts of China, leading to a wider epidemic, or it may lead to more serious epidemic status of paratuberculosis in other regions.

To evaluate the risk of cattle infected with MAP in different scale farms, we classified all farms into small and large farms. We found that the probability of cattle in small farms being infected with MAP was about three times higher compared with large farms. According to previous studies, many factors can lead to paratuberculosis transmission; e.g., the general indoor environment is the most critical transmission route followed by in-utero transmission [36]. The farms that buy and sell dairy cattle also have the highest infection risk for MAP infection [37]. Furthermore, the attitudes of farmers towards MAP and their beliefs are very important for the control of MAP [38]. In China, farmers in small farms usually have a relatively low knowledge and a negative attitude towards the control of animal diseases, and most of them buy dairy cattle from others. As we mentioned above, MAP is a easily ignored disease in China, because, to our knowledge, almost no farms test new animals for paratuberculosis. In large farms, although they also do not care about the surveillance and detection of paratuberculosis, farmers pay much more attention to the biosafety of the farms, including disinfection, not buying animals from high-risk areas, and the training of farm workers. This attention may be one of the most important reasons why small-scale farms have a higher risk of getting paratuberculosis than large-scale farms. Nevertheless, it needs further investigation. 

Based on a comprehensive range of scientific findings, it is becoming progressively evident that a noteworthy correlation exists between milk production, milk quality parameters, and bovine paratuberculosis. In this study, we continue our research to determine whether cows with different antibody levels would exhibit variations in milk quality. Viktor Jurkovich et al. showed that in high-risk cows there were significant losses of milk, protein, and fat yields and higher SCCs [39], and these conclusions almost align with our study. Undoubtedly, milk production is the most concerning issue for farmers. In this study, we reported that the average milk yield of test-positive cows was 21.1 ± 9.7 kg/day, a reduction of about 14.2% compared to test-negative cows (24.6 ± 11.2 kg/day) (Table 5). This aligned with a reported 15–16% decrease in milk production [12], which leads to huge economic losses. U.S. Sorge et al. also concluded that paratuberculosis can decrease milk production, with ELISA-positive cattle exhibiting a 2.9% to 6.8% reduction compared to ELISA-negative cattle [8]. Lactose is a significant issue for the solids content of many dairy products and plays an important role in the determination of the properties of milk products [40]. Our findings (Table 5) revealed that the cows with higher antibody levels demonstrated a significantly (*p* < 0.05) lower lactose content, which caused a decline in milk quality. The somatic cell count (SCC) in milk often be used as an indirect marker for mastitis infections in the dairy breeding. An SCC of more than 100,000 cells/mL is commonly suggestive for a subclinical mastitis infection [41]. Laszlo Ozsvari et al. reported that milk ELISA-positive cows had a somatic cell count that was an average of 35.8% higher than test-negative cows [42]. In this study, we also found that MAP-positive cows had a somatic cell count that was an average of 53.9% higher than test-negative cows (*p* < 0.05). Gonçalves reported that, during lactation, adults cows with an average of 500,000 SCC/mL can lose 3.04 kg of milk/day, corresponding to 927 kg of milk during lactation for Holsteins cows [43]. Although we do not know whether paratuberculosis causes mastitis or whether mastitis is responsible for MAP, it can be confirmed that there is a strong correlation between the occurrence of paratuberculosis and mastitis. The coexistence of those two diseases will greatly reduce milk production and quality. The milk fat content percentage, total solids percentage, and urea nitrogen were not statistically significant in our study. 

All these findings provide compelling evidence that bovine paratuberculosis is endemic in China and can lead to significantly economic losses. As a result, paratuberculosis cannot be ignored in China and urgently needs to be prevented and controlled. 

## 5. Conclusions

This study’s results corroborate the idea that paratuberculosis testing in both milk and blood shows a high degree of agreement, suggesting that both methods might be used effectively to track the spread of the illness among cattle. Paratuberculosis has a high prevalence and incidence risk in Hubei Province. Furthermore, the disease leads to a decrease in milk production and the lactose content percentage and an increase in the milk protein percentage and SCC in Hubei Province dairy cows. However, further research is required to substantiate this phenomenon and to understand the underlying mechanisms. This highlights the need for prompt control measures to mitigate the spread of the disease. 

## Figures and Tables

**Table 1:** Location, scale, and sampling information from the farms where the samples were collected.

City	Farms	Scale	January 2018		April 2018	
			No. of Lactating Cows	No. of Samples Tested	No. of Lactating Cows	No. of Samples Tested
WH	A	Large	1321	142	1121	99
	B	Small	94	77	97	77
HG	C	Small	N/A	N/A	146	146
	D	Small	100	80	99	78
	E	Large	1092	274	1596	104
	F	Large	1068	201	1108	170
	G	Small	267	156	272	92
	H	Small	191	126	180	105
	I	Large	1883	156	1717	86
	J	Small	140	140	150	92
	K	Small	57	57	84	51
	L	Small	131	96	124	75
YC	M	Small	117	91	128	81
Total	13		6682	1596	6876	1256

**Table 2:** A 2 × 2 table of the milk and serum ELISA results for paratuberculosis on three farms in Hubei Province, China.

		Serum Antibody Test		
		Positive	Negative	Total
Milk antibody test	Positive	22	4	26
	Negative	8	115	123
	Total	30	119	149

**Table 3:** Paratuberculosis prevalence in milk samples tested by ELISA on farms in Hubei Province, China.

Farm	January 2018		April 2018	
	Prevalence (%)	95% CI	Prevalence (%)	95% CI
A	2.1	0.4, 6.0	0.0	0.0, 3.7
B	18.2	10.3, 28.6	14.3	7.4, 24.1
C	N/A	N/A	19.9	13.7, 27.3
D	18.8	10.9, 29.0	11.5	5.4, 20.8
E	2.9	1.3, 5.7	1.9	0.2, 6.8
F	3.5	1.4, 7.0	1.8	0.4, 5.1
G	1.3	0.2, 4.6	0.0	0.0, 3.9
H	7.9	3.9, 14.1	9.5	4.7, 16.8
I	9.0	5.0, 14.6	17.4	10.1, 27.1
J	17.1	11.3, 24.4	17.4	10.3, 26.7
K	15.8	7.5, 27.9	17.6	8.4, 30.9
L	22.9	15.0, 32.6	18.7	10.6, 29.3
M	4.4	1.2, 10.9	6.2	2.0, 13.8
Large farm	4.1	2.8, 5.8	4.4	2.7, 6.6
Small farm	12.2	10.0, 14.6	12.9	10.7, 15.5
Total	8.3	7.0, 9.7	9.8	8.2, 11.6

**Table 4:** Incidence risk of paratuberculosis according to the milk sample ELISA results on farms in Hubei Province, China.

Farms	Numbers of Cows That Tested Negative then Positive	Numbers of Cows That Tested Negative at the Beginning	Incidence Risk (/3 months) (%)	95% CI
A	0	97	0	0.0, 3.7
B	N/A	N/A	N/A	N/A
C	N/A	N/A	N/A	N/A
D	5	33	15.2	5.1, 31.9
E	1	60	1.7	0.0, 8.9
F	1	123	0.8	0.0, 4.4
G	0	89	0	0.0, 4.1
H	6	96	6.2	2.3, 13.1
I	7	46	15.2	6.3, 28.9
J	11	74	14.9	7.7, 25.0
K	4	43	9.3	2.6, 22.1
L	5	61	8.2	2.7, 18.1
M	8	78	10.3	4.5, 19.2
Small farms	39	474	8.2	5.9, 11.1
Large farms	9	326	2.8	1.3, 5.2
Total	48	800	6	4.5, 7.9

**Table 5:** Comparison of milk yield composition in cows with a different paratuberculosis status.

Antibody Test Results	Milk Yield (kg/day)	Fat Content Percentage (%)	Milk Protein Percentage (%)	Lactose Content (%)	Total Solids Percentage (%)	SCC (×1000 cell/mL)	Urea Nitrogen Percentage (%)
negative (*n* = 2597)	24.6 ± 11.2	3.9 ± 1.5	3.4 ± 0.4	5.0 ± 0.3	12.8 ± 1.5	413.8 ± 1264.4	14.1 ± 2.7
95% CI	24.2, 25.1	3.8, 3.9	3.37, 3.4	5.0, 5.01	12.7, 12.9	365.1, 462.4	14.0, 14.2
positive (*n* = 255)	21.1 ± 9.7	4.1 ± 1.9	3.6 ± 0.8	4.8 ± 0.5	13.0 ± 2.1	898.1 ± 2491.2	14.3 ± 4.6
95% CI	19.9, 22.2	3.9, 4.3	3.5, 3.7	4.8, 4.9	12.8, 13.3	590.8, 1205.3	13.7, 14.8
*p* value	0.000	0.057	0.001	0.000	0.083	0.002	0.49

## Data Availability

All data are contained within this article.
